# Supplementary figures and images for: Non-linear auto-regressive models for cross-frequency coupling in neural time series
Source: PLoS Comput Biol. 2017 Dec 11;13(12):e1005893. doi: 10.1371/journal.pcbi.1005893 (PMC5739510; doi:10.1371/journal.pcbi.1005893)

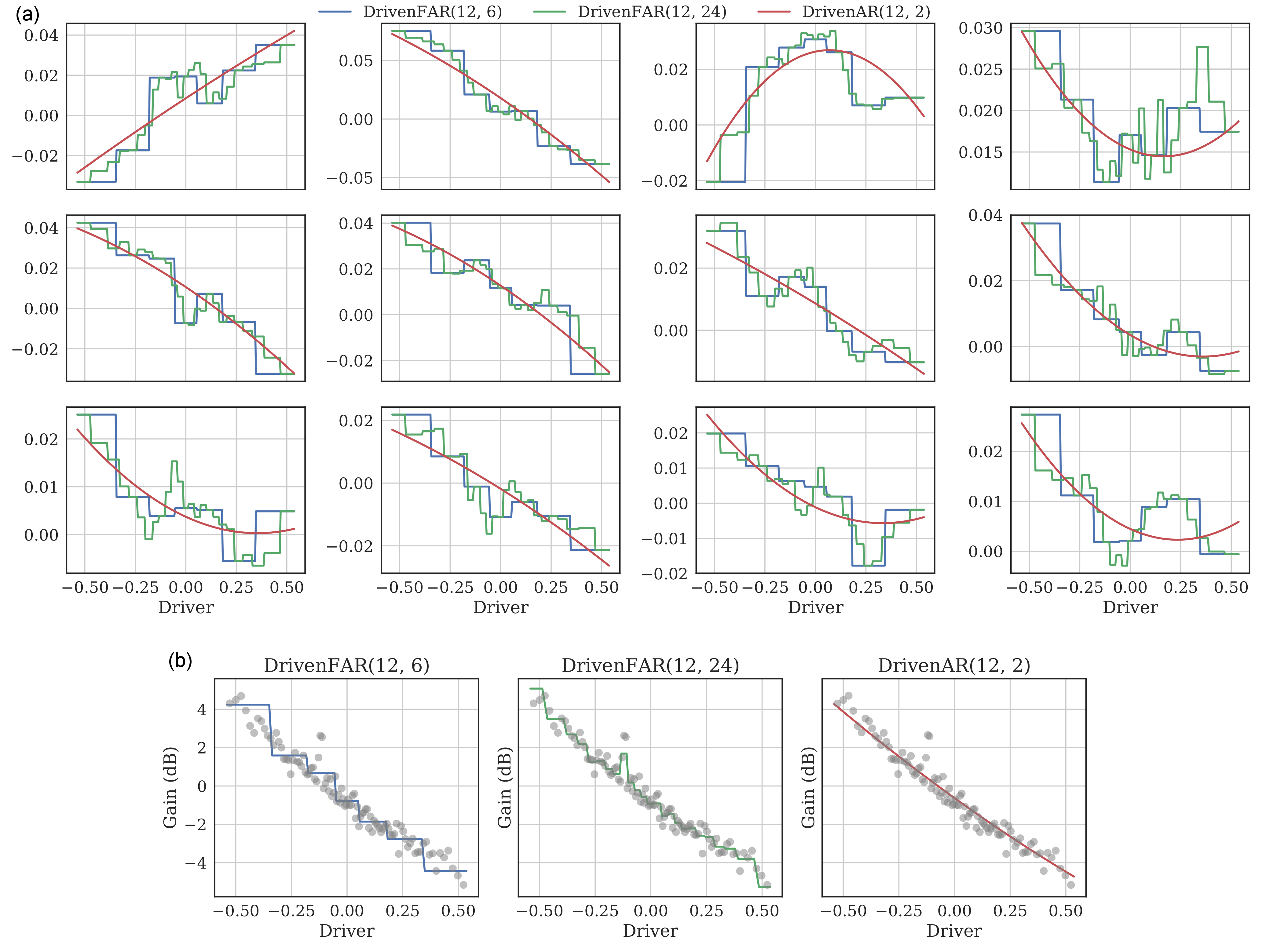

Supplement: S1 Fig — (a) AR coefficients ai(x) as functions of the driver x. (b) Innovation variance σ2(x) as functions of the driver x. The gray circles are the mean squared-residual over 100 bins of the driver values. We compared three parametrizations of these functions: Two staircase functions with respectively s = 7 and s = 25 steps, and one polynomial function with order m = 2, as used in the rest of this work. For a piecewise constant AR parametrization with s-steps, we divided the driver’s values into s equally distributed bins, then we fitted s independent linear AR models on the time samples of each bin. This approach is more general than the polynomial approach, yet each AR model uses only T/s samples, whereas the polynomial approach in the DAR model uses all the T samples. The DAR model is therefore more robust. The polynomial parametrization also uses fewer coefficients since a low order m is sufficient. The models are fitted on the human cortical signal, using p = 12. We see that an order-2 polynomial is sufficient to approximate the trend of both the AR coefficients and the innovation variance, as estimated by the staircase functions, yet the polynomial uses much fewer parameters. (TIF) [file pcbi.1005893.s001.tif]

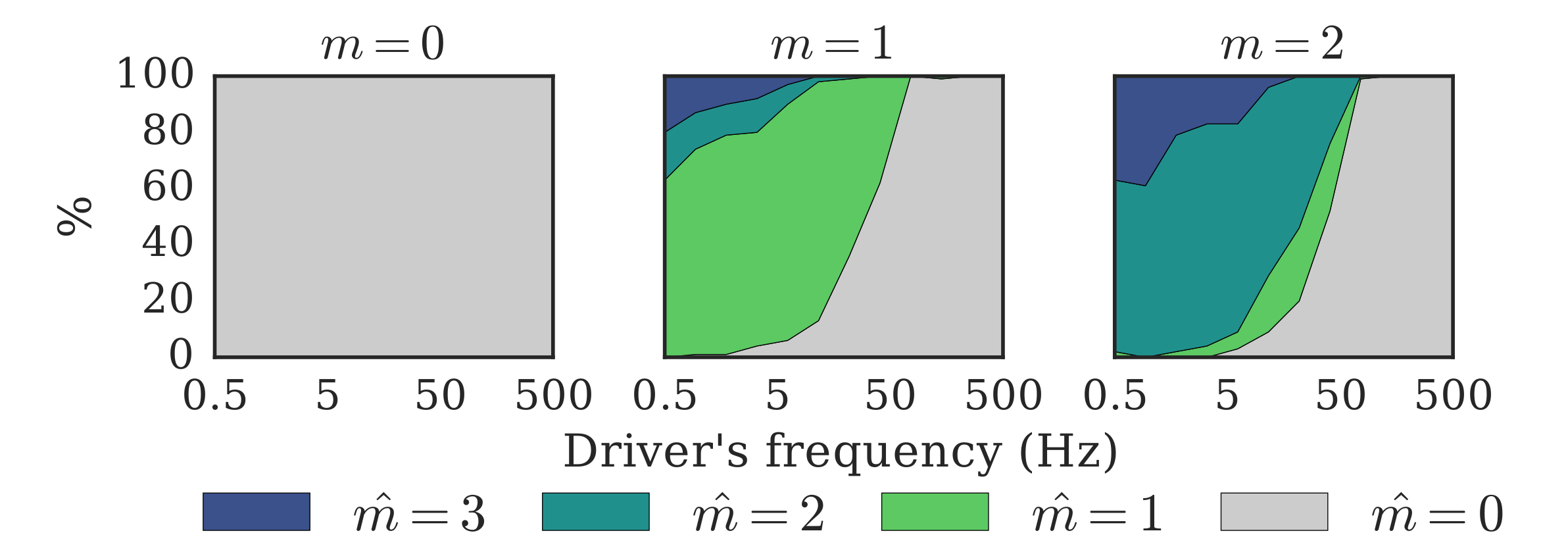

Supplement: S2 Fig — We simulated 100 signals from 100 DAR models with p = 10 and m ∈ [0, 1, 2]. We then estimated new DAR models on these signals, with p ranging from 1 to 20, and m ranging from 0 to 3. We selected p^ and m^ that minimized the BIC. The graphs show the proportion of runs that lead to each value of m^. The hyper-parameter m is correctly estimated in most cases if the driver’s frequency is not too high (fx < 50 Hz). The hyper-parameter p is correctly estimated at ±2 in most cases. (TIF) [file pcbi.1005893.s002.tif]

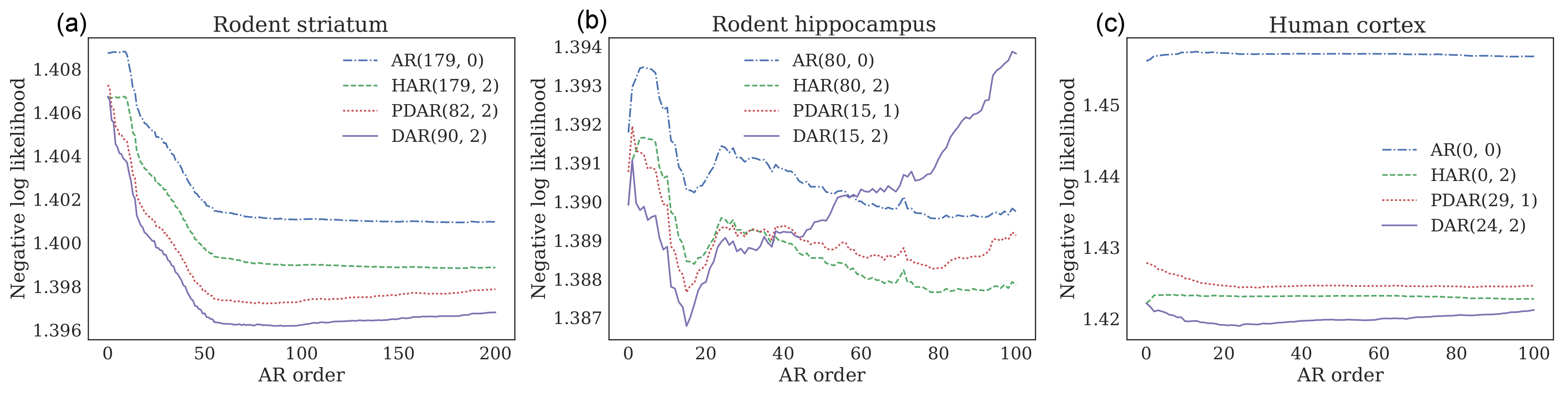

Supplement: S3 Fig — Cross-validation on our three dataset, (a) rodent striatum, (b) rodent hippocampus, and (c) human auditory cortex, to select the best model and the best parameters. Splitting the signal in half, we fitted the models on the first half, and evaluated the model likelihood on the second half. We compared four different models on a grid of parameter p ∈ [0, 100–200] and m ∈ [0, 3]: AR: a linear AR modelHeteroskedastic AR (HAR): an hybrid model between a linear AR model and a DAR model, where the innovation variance σ2 is driven by x, but the AR coefficients are constant in time.Phase DAR (PDAR): a DAR model, with a normalized driver: x/|x|. In this way, we only consider the phase of the slow oscillation, as in most PAC metrics.DAR: a DAR model, where both the innovation variance σ2 and the AR coefficients are driven by x. The figures present the negative log likelihood (lower is better) by time sample. Each line corresponds to a given model with its best parameter m. The legend shows which orders (p, m) are the best for each model. One can observe that the curves of negative log-likelihood are not convex, yet they exhibit rather clear minima used to define the optimal paramaters. (TIF) [file pcbi.1005893.s003.tif]

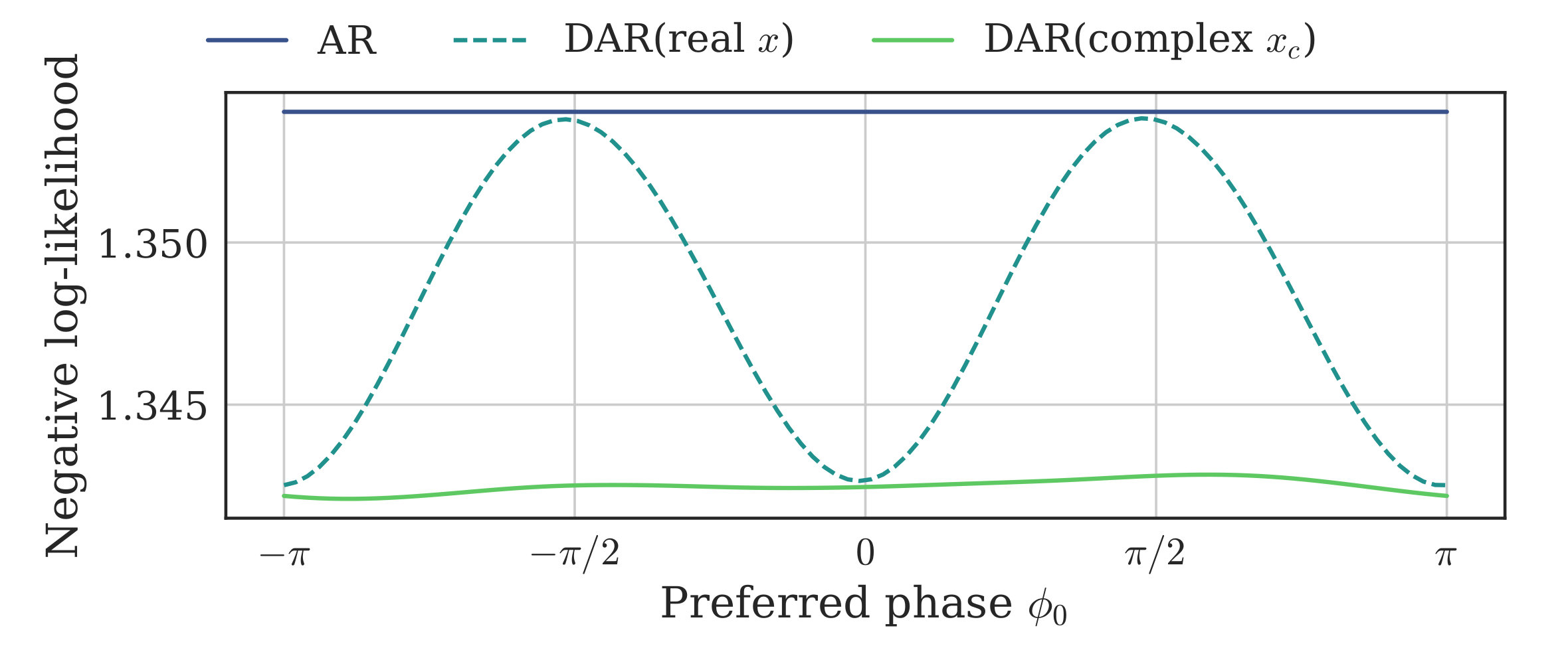

Supplement: S4 Fig — We simulated a signal as described in the Methods section, introducing a phase difference ϕ0 in the modulation. For each value of ϕ0, we fitted three different models, and compared their negative log-likelihood by time sample (the lower the better): an AR, a DAR with a real driver, and a DAR with a complex driver. The parameters were set to p = 10 and m = 1. A bias is visible around ϕ0 = ±π/2, since the real-valued driver DAR model does not fit better than the AR model. As expected, this bias disappears when we update the model to a complex-valued driver DAR model. (TIF) [file pcbi.1005893.s004.tif]
